# How accurate is patients' anatomical knowledge: a cross-sectional, questionnaire study of six patient groups and a general public sample

**DOI:** 10.1186/1471-2296-10-43

**Published:** 2009-06-12

**Authors:** John Weinman, Gibran Yusuf, Robert Berks, Sam Rayner, Keith J Petrie

**Affiliations:** 1 Health Psychology Section, Psychology Dept, Institute of Psychiatry, KCL, 5th floor Bermondsey Wing, Guy's Campus, London Bridge, London, SE1 9RT, UK; 2 Department of Psychological Medicine, Faculty of Medicine and Health Sciences University of Auckland, Private Bag 92019, Auckland, New Zealand

## Abstract

**Background:**

Older studies have shown that patients often do not understand the terms used by doctors and many do not even have a rudimentary understanding of anatomy. The present study was designed to investigate the levels of anatomical knowledge of different patient groups and the general public in order to see whether this has improved over time and whether patients with a specific organ pathology (e.g. liver disease) have a relatively better understanding of the location of that organ.

**Methods:**

Level of anatomical knowledge was assessed on a multiple-choice questionnaire, in a sample of 722 participants, comprising approximately 100 patients in each of 6 different diagnostic groups and 133 in the general population, using a between-groups, cross-sectional design. Comparisons of relative accuracy of anatomical knowledge between the present and earlier results, and across the clinical and general public groups were evaluated using Chi square tests. Associations with age and education were assessed with the Pearson correlation test and one-way analysis of variance, respectively.

**Results:**

Across groups knowledge of the location of body organs was poor and has not significantly improved since an earlier equivalent study over 30 years ago (χ^2 ^= 0.04, df = 1, ns). Diagnostic groups did not differ in their overall scores but those with liver disease and diabetes were more accurate regarding the location of their respective affected organs (χ^2 ^= 18.10, p < 0.001, df = 1; χ^2 ^= 10.75, p < 0.01, df = 1). Age was significantly negatively correlated (r = -0.084, p = 0.025) and education was positively correlated with anatomical knowledge (F = 12.94, p = 0.000). Although there was no overall gender difference, women were significantly better at identifying organs on female body outlines.

**Conclusion:**

Many patients and general public do not know the location of key body organs, even those in which their medical problem is located, which could have important consequences for doctor-patient communication. These results indicate that healthcare professionals still need to take care in providing organ specific information to patients and should not assume that patients have this information, even for those organs in which their medical problem is located.

## Background

Communication by doctors in medical consultations often assumes that the patient has basic knowledge of the body and its functioning. However, a number of studies have shown that patients do not understand the terms used by doctors and many patients do not even have a rudimentary understanding of anatomy. Studies show that a large percentage of patients do not know the difference or similarity between pairs of medical terms (e.g. heart-attack and myocardial infarction; fracture and broken bone) [1]. These basic misunderstandings could have direct effects in the consultation since doctors may use anatomical and other technical terms under the mistaken belief that these will be readily understood by their patients. This overestimation of patient knowledge has been shown to have negative effects on doctor-patient communication in a range of healthcare settings [2].

An early study of hospital outpatients and doctors by Boyle showed that the public awareness of the anatomical location of key body organs was quite poor, particularly when compared with the level of doctors' performance. Using a multiple-choice measure with 4 body outlines each indicating a possible location of an organ, Boyle showed that the location of eight key body organs was correctly identified approximately fifty percent of the time [3]. Other research shows that, even in patients with specific organ-related disorders, their knowledge of the location of that particular organ was poor. For example Pearson and Dudley [4] found only 12% of gastrointestinal patients were able to identify correctly the location of the affected organ. Such discrepancies in anatomical knowledge between doctors and patients can have significant effects in the consultation, and on subsequent patient satisfaction and adherence [5,6]. For example, not only have these discrepancies been shown to give rise to misunderstandings in the consultation [5] but also when specific attempts were made to remedy these then this resulted in greater patient satisfaction with the consultation on a number of important dimensions [6].

Since Boyle's study almost 40 years ago there have been a number of societal changes that may have improved the public's level of medical knowledge. There have been improvements in education, coupled with an increased media focus on medical and health related topics, and growing access to the internet as a source of medical information [7]. We therefore felt it was timely to assess again the public's level of anatomical knowledge and to extend the original study to investigate whether patients with a specific organ pathology (e.g. liver disease) have a relatively better understanding of the location of that organ.

## Methods

A cross-sectional questionnaire-based design was adopted. Anatomical knowledge was the dependent variable. Independent variables were clinical group, gender, age, occupation, age on leaving fulltime education, clinical experience, and internet usage for medical information. The study was approved by the Guy's & St Thomas' Hospitals and King's College Hospital Ethics Committees.

In total there were 722 participants, 589 of which were from each of 6 clinical groups (cardiac n = 103, respiratory n = 97, renal n = 102, liver n = 97, diabetes n = 95, gastrointestinal n = 95) and 133 were also collected from the general population. The patient samples were drawn from out-patient departments in Guy's, King's and St Thomas's hospitals, and the general population was an opportunistic sample obtained primarily from users of a south London public library and was broadly matched in socio-demographic characteristics to the patient groups. Thus the mean age and (percentage of males) in each group was as follows: cardiac = 56.3 years (52% Male); respiratory = 46 years (45% M); renal = 49.5 years (59% M); liver = 47.8 years (53% M); diabetes = 54.6 years (46% M); gastrointestinal = 47.4 years (55% M); general population = 47.1 years (46% M). Each group in this sample is also comparable to that used by Boyle since this consisted of 114 mixed outpatients with a mean age of 45 years and a male:female ratio of 2:3. The sample sizes were determined to allow us to detect a medium effect size difference between study groups at 80% power and a significance level of .05, based on previous findings.

**Figure 1:**
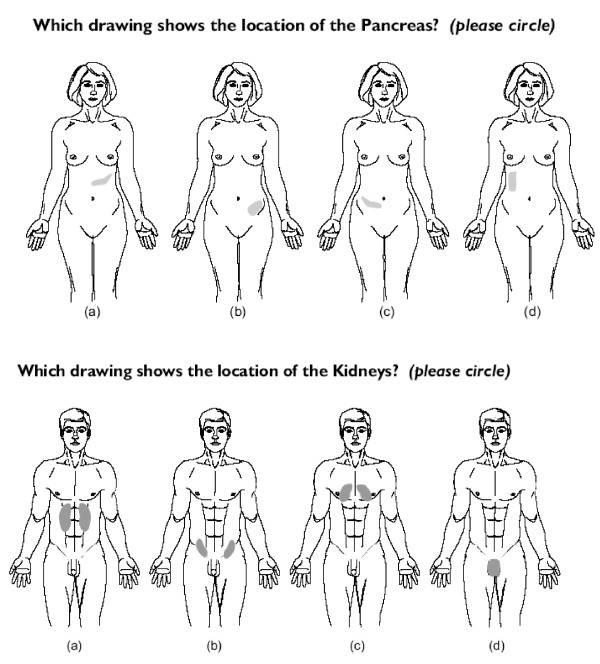
**Two examples of items used for assessing anatomical knowledge**.

After obtaining verbal consent, participants were given the questionnaire, which they were asked to complete at the time on their own. The first part of the questionnaire consisted of basic socio-demographic questions, which were followed by 11 items testing knowledge of the location of a specific body organ. Each of these showed body outlines with the organ in four possible locations, and the participant was asked to select the one they felt was correctly located (see figure 1). Eight of these items (heart, lungs, stomach, intestines, bladder, thyroid, liver, kidneys) replicated Boyle [3] and there were 3 additional items (pancreas, gallbladder, ovaries) which used a female body outline (see figure 1). Although not formally tested for their psychometric properties, these items were selected to replicate those previously used [3] and in each group there was a spread of responses across each of the four choices. Moreover these simple ways of assessing anatomical and other medical knowledge have been shown to have good reliability and validity [8].

The data were entered into SPSS 12 for statistical analysis. Comparisons of the relative accuracy of anatomical knowledge between the present and earlier results, and across the clinical and general public groups were evaluated using Chi square tests. Associations with age and education were assessed with the Pearson correlation test and one-way analysis of variance (ANOVA), respectively.

## Results

To assess whether patients with problems which affected specific organs were more accurate in their knowledge of the location of that organ, comparisons were made across all groups for each organ and the results are shown in table 1.

**Table 1:** Percentages of correct answers for the location of each organ by each clinical group and the general public sample (*means shown in bold indicate significantly better performance than the general public sample*).

	**General Public**	**Respiratory**	**Renal**	**Cardiac**	**Liver**	**Diabetes**	**Gastro-intestinal**
**1. Liver**	45.9	56.7	45.1	53.4	***75.3***	45.3	51.6

**2. Ovaries**	39.8	38.1	36.3	30.1	42.3	28.4	42.1

**3. Heart**	55.6	41.2	42.2	50.5	44.3	44.2	44.2

**4. Thyroid**	36.1	42.3	52.9	60.2	39.2	35.8	26.3

**5. Lungs**	27.1	37.1	27.5	32.0	30.9	34.7	32.6

**6. Gallbladder**	32.3	37.1	32.4	38.8	40.2	37.9	26.3

**7. Pancreas**	30.8	36.1	29.4	37.9	33.0	***53.7***	29.5

**8. Intestines**	94.0	86.6	78.4	84.5	86.6	86.3	82.1

**9. Bladder**	85.0	84.5	81.4	76.7	82.5	74.7	78.9

**10. Kidneys**	27.1	50.5	42.2	49.5	37.1	41.1	55.8

**11. Stomach**	48.1	43.3	28.4	31.1	37.1	31.6	46.3

The only clinical groups correctly identifying their affected organ significantly better than the general population were patients with liver disease (χ^2 ^= 18.10, p < 0.001, df = 1) and diabetes (χ^2 ^= 10.75, p < 0.01, df = 1).

To assess whether overall anatomical knowledge had improved since 1970, the responses for the total sample were compared with the data from Boyle's earlier study and the results are shown in table 2.

**Table 2:** A comparison of the percentage correct for each item and for the total in Boyle(1970) and the present study.

	Heart	Lungs	Stomach	Intestines	Bladder	Thyroid	Liver	Kidneys	MEAN TOTAL (s.d)
Boyle % correct	43.6	50.9	20.2	76.9	59.8	69.9	48.5	42.5	51.4 (17.7)

Present study % correct	46.5	31.4	38.4	85.9	80.7	41.8	52.9	42.5	52.5 (20.1)

No overall difference was found in the percentage correct between the two samples (χ^2 ^= 0.02, df = 1, p < 1), although there were some differences in the accuracy of knowledge concerning the location of specific organs.

A number of socio-demographic factors were also found to be associated with anatomical knowledge. Across all groups, age showed a small but significant negative correlation (r = -0.084, p = 0.025) and education showed a highly significant effect with more educated participants being more accurate (F = 12.94, p = 0.0001). Although there was no significant gender difference in overall anatomical knowledge (see table 3), women scored significantly higher on the three items, which used a female body outline (ovaries: χ^2 ^= 9.7, p = 0.00); gallbladder: χ^2 ^= 8.67, p = 0.001; pancreas; χ^2 ^= 18.27, p = 0.001).

**Table 3:** Gender comparison of the percentage correct responses for each question and the overall averages.

% correct	Liver	Ovaries	Heart	Thyroid	Lungs	Gallbladder	Pancreas	Intestines	Bladder	Kidneys	Stomach
Male	51.2	31.7	46.1	41.3	29.9	30.1	28.3	87.2	79.5	40.5	33.6

Female	55.0	43.0	47.1	42.7	32.7	40.6	43.6	84.5	82.2	45.0	43.9

OVERALL AVERAGE: Male – 45.4% (s.d = 20.2); Female – 50.9% (s.d.= 16.9)

## Discussion

The present study investigated the ability of lay people to identify the correct anatomical location of key body organs and found that overall levels of knowledge were very similar to that found almost 40 years ago [3]. Perhaps even more surprising is the level of anatomical knowledge in patients with organ-specific pathology since only two of the six clinical groups in the current study showed better anatomical knowledge of the location of their affected organ.

Even with increased media focus on health and widespread availability of health information on the internet [7], the lack of any improvement over the years probably reflects the fact that there has been no systematic attempt to promote access to this type of knowledge. Other studies since Boyle's, using different methodologies, have also revealed equivalent discrepancies in levels of medical and lay anatomical knowledge [4,9,10]. However none of these have attempted to investigate systematically whether knowledge of the location of specific organs would be better in patients with disorders affecting that organ.

The very specific finding that women's anatomical knowledge was superior to men's when a female body outline was used clearly merits further exploration, particularly with a more balanced study design to assess these effects systematically. It therefore emphasizes the need for incorporating a balance of male and female outlines in future studies assessing anatomical knowledge.

Even though the present study used a much larger sample than the original study, and involved both general public and specific patient groups, there are still some limitations in the sampling procedure. The general public sample was primarily taken from a public library, and the clinical samples were taken opportunistically from outpatient settings. While the groups were reasonably well-matched for age and gender balance, they were only broadly matched for educational experience. Although the multiple-choice method for assessing anatomical knowledge was based on that used in the original study [3] and has been used a number of times since then, it would benefit from formal psychometric evaluation to establish its reliability and validity.

The study has a number of implications for doctor – patient communication and for both medical and health education. Following Boyle's study, concern has been expressed about the potential problems, which these sorts of findings could have for doctor-patient communication, with possible adverse effects on diagnosis and treatment outcomes [5]. Some problems of communication may well be reduced by the doctor and patient being able to point to affected areas, rather than having to rely completely on the organ name to verify the location of a problem. Nevertheless, the implications of discrepant anatomical understanding have been explored in a number of studies of doctor-patient communication [11,12] and negative effects have been found on patient understanding and satisfaction following the consultation. Moreover recent evidence shows that when doctors' and patients' vocabulary for anatomical and other terms are matched in the consultation, then significant gains are found in patients' overall satisfaction with the consultation as well as with specific components of it such as rapport, communication comfort and compliance intent [6].

## Conclusion

These results indicate that the ability of patients and lay people to identify the correct anatomical location of key body organs is still quite limited. Healthcare professionals still need to take care in providing organ specific information to patients and should not assume that patients have this information, even for those organs in which their medical problem is located. The consultation may offer many opportunities for both checking and improving patients' knowledge.

## Competing interests

The authors declare that they have no competing interests.

## Authors' contributions

JW designed and supervised the study, and drafted the manuscript. GY, RB and SR collected and analysed all the data. KP participated in the study design and helped to draft the manuscript

## Pre-publication history

The pre-publication history for this paper can be accessed here:


